# Æsculapian Society of the Wabash Valley

**Published:** 1871-09

**Authors:** Wm. M. Chambers, G. T. Ragan

**Affiliations:** President; Secretary


### Proceedings of Societies.

### ÆSCULAPIAN SOCIETY OF THE WABASH VALLEY.

THE Semi-annual Meeting of this Society convened at Effingham, Ill., May 31st, 1871, Dr. Wm. M. Chambers, president, in the chair; Dr. R. L. Wallston, of Paris, Secretary *pro tem.*, read the proceedings of the Annual Meeting, when Dr. G. T. Ragan, of Neoga, was selected as Secretary for the remainder of the session.

After the usual business in form and the discussion of some subjects not of general interest, Dr. C. B. Cannon, chairman of Committee on Surgery, read a very interesting paper, especially written to give the author's views in regard to his experience in the use of the hypodermic injection of morphia as a curative agent in disease. His attention was called to this method in the treatment of sciatica and delirium tremens in an article in *Braithwait's Retrospect*, in 1861. Shortly afterwards commenced using it, but found the facts given in *Braithwaite* were not fully verified by personal observation, such as obtaining relief and sleep in five minutes after injecting one grain of morphia. It was often fifteen or twenty minutes, and then instead of quiet sleep, lasting from twelve to twenty hours, there was wakefulness with troublesome vomiting. This discrepancy was attributed to the physical condition of the patients. In hospital, the patients had been reduced in flesh, worn out by pain, physicked and dosed with all the usual remedies for sciatica before the injections were used. In the Doctor's private practice, his patients had no preparation whatever, but when he did prepare them uniform success followed, and he now feels free to say that any case of idiopathic neuralgia, wherever it may be located, will yield, after the proper regulation of the bowels, to the hypodermic exhibition of morphia in full doses; would use from two-thirds to one grain of the salt, according to the condition and habits of the patient, sufficient to produce narcotism, and make a profound impression on the nervous system. Ordinary idiopathic neuralgia, dum ague, sun pain, from malarial causes, will yield to a single administration. No treatment will be successful in neuralgia dependent upon nervous irritation, caused by spicula of bone or ulcerated tooth, until the exciting cause is removed. Cases complicated with other diseases must be treated in accordance with the complications. Has never seen a case of gout, but has little doubt that after the preparatory treatment and the administration of colchicum, the nervous impression made by narcotism, induced by injection, would stop the paroxysm. Reported a case of a lady aged seventy, had been suffering from facial neuralgia for five years, had been treated by regular physicians and travelling quacks without any permanent relief.

The teeth all having been removed from that side without any salutary effect, the Doctor removed a portion of the jawbone, followed by tonics and anodynes, still only temporary relief; as a last resort, determined to use the injections; and accordingly, localized a full portion of morphia near the seat of the pain. The patient soon passed into a sleep that lasted through the afternoon and following night, she awoke next morning free from pain, and since that time has never had a single paroxysm and is fully recovered. Had used the injections several times in delirium tremens with very unsatisfactory results.

The want of success may be attributed to two causes: First —The extreme tolerance in this disease of the nervous system to the effects of opium; and secondly, that many of the so-called cases of delirium tremens, especially the fatal ones, are really the result of organic lesion within the brain, and the connection between the sensorium and the ultimate ramifications of the nerves is so modified by disease as to render powerless any influence to produce sleep. Remarks were made by Drs. Miller, Todd, and Chambers in support of Dr. C.'s views. Dr. Chambers thinks the solution should be recent, it loses its virtue by age; he never goes abroad without his hypodermic apparatus, prefers the sulphate to the acetate of morphia.

Dr. Wallston read the report upon obstetrics which elicited a general discussion. Dr. L. J. Willien reported a case of ulceration of the neck of the uterus of five years' standing. Mrs. L. Z., aged 22, nervous lymphatic temperament, light complexion—large—had menstruated for the first time at the age of fifteen, painful menstruation ever since, married at seventeen, general health good till after marriage, when menses were suppressed and replaced by a white mucus discharge which gradually became a constant flow, and lastly, becoming of a pasty and yellow-green color. This continued till 1867, one year after marriage, when a high state of chlora anæmia existed, with discoloration of conjunctiva and lips, fauses pale, extremities cold, vertigo, palpitations of the heart, with a clear bellows-sound extending along the carotids anorexia, and constipation; neuralgic pain darting to and from the chest, and then through the lower part of the bowels. At first sight supposed the uterus to be the seat of the disease, but patient not consenting to an examination, ordered antispasmodics, ecbolics, tonics, such as cinchonia and iron, good diet, and astringent injections,—temporary relief followed. March, 1871,—was called to see her; the general symptoms of debility having increased; this time, having become wiser, she consented to a vaginal exploration with the speculum; the following condition was discovered:

1st. Engorgement of the cervex uteri.

2nd. Ulceration of the anterior labia extending into the os.

3rd. Abundant muco-purulent discharge from the os; this being very acid, as shown by the application of blue litmus paper. The external organs were pale, relaxed, and also the mucous membrane of the vigina.

Therefore, determined to submit the patient to a local and general treatment. After removing all mucosities from vagina and neck of the womb, made a free application of lunar caustic to the ulcer, and into the cavity as far as could reach, and applied a small cotton plug, imbibed with a mixture of carbolic acid twelve drops to glycerine one ounce, the plug to be removed next morning, and followed by a weak solution of bicarb. of soda. On the third day, found the discharge more abundant, but of a thinner character, the ulcer appeared red and granulating; lunar caustic was again applied and dressed as before.

Tenth day, the patient says she is improving, appetite returns and the pain in the lower part of the abdomen is less severe. Fifteenth day, the ulcer is disappearing and replaced by healthy granulations. Caustic is omitted and carbolized glycerine injected into the cavity of the neck and the plug applied. On the seventeenth day, after sleeping well during the night, she awoke, and found to her great surprise her menses had appeared, not evincing the least pain or uneasiness; this lasting five days, after which time, the leucorrhœal discharge subsided rapidly, the ulcer healed, and the engorgement has entirely disappeared. Twice weekly dressed the parts, using the astringent lotion, *viz.*:

℞. Sulph. Copper, ---------------------- ʒj.

Tannin, ---------------------------- ʒij.

Rose Water, ---------------- --------- ℥j.

Carbolic Acid, ------- ---------- gtt., xvi.

Rain Water, ---------------- -------- ℥ij.

Sig. Imbue the cotton plug with this solution, or if the orifice permits, inject or apply with camel hair brush.

This to be used every day internally to calm nervous hyperæsthisia:

℞. Hydrate of Chloral, ------------------ ʒij.

Elix. Val. Ammonia, ------------------ ℥j.

M. ʒj every hour or two.

As a tonic and alterative.

℞. Iodide Potass., ----------------------- ʒiv.

Elix. Protox. Iron and Cinch., ------- ℥viii.

Tinc. Rhei, ------------------------- ℥ij.

M. Dessert spoonful four times a day.

To assist the digestive organs, create appetite and overcome constipation, the following pill:

℞. Ext. Nux Vomica.
" Belladonna.
" Stramonii.
" Conii Mac.
" Rhei, āā gr. vi.
Pul. Aloes locot, gr. xxiv.

M. Ft. Pil. No. xxiv, one pill ter die one hour before meals.

This prescription against any scrofulous or malarial tendency.

℞. Syr. Ferri Iodide, .................... ℥j.

Solution Fowleri,................... ʒijss.

Aq. Menth. Pip.,.................... ℥ij.

Sig. Teaspoonful three times a day, one hour before meals. Also ordered lotions along the spine and abdomen with good whiskey and dry friction on the skin. May 11th, patient enjoys good health, and all symptoms of anæmia have subsided.

Dr. L. L. Todd reported a case of eclampsia treated successfully with chloroform and hypodermic injection of acet. of morphia. Would use the injections again under similar circumstances.

An animated discussion on the arrest of hemorrhage in placenta previa was participated in by a number of the members, for and against turning, and the use of the tampon. The objection, on the part of some, to the use of the tampon seemed to result from a misconception of the philosophy of its application.

Dr. J. H. Apperson reported a case of peculiar condition of adherent placenta, termed by him, fibrous tumor adherent, and in common with placental mass. *Post mortem* revealed the fact, that the tumor, uterus, and placenta were inseparable, the union being so complete. Dr. Todd thought it a case of fibroid degeneration of placenta.

Dr. Wm. Massie made a verbal report of a case of senile gangrene, patient eighty-two years of age, infirm, constitution dyspeptic. One year ago, last Feb., his nails on left foot hurt him,—pared them closely, became very painful, lost several nights' sleep. The Doctor found no cause for pain, except from paring the nails. After several days, a dark spot appeared, this sank and left an eschar, then an ulcer. He wore a moccasin all summer, foot cold all the time. In Feb. of this year, horse trampled on this foot, divided a small place over the metatarsus. Whenever the foot was warmed, it gave him great pain. He commenced losing flesh; called on the Doctor; began treatment to stimulate action; the flesh sloughed to the bone. Both feet became swollen, also face and hands, went down rapidly, then seemed to rally. In two weeks toe began to mortify, the next toe began the same way, not in connection with the former. One after another went the same way, till all died, when the foot began; sometimes the mortification would stop for three or four days, when ease would follow, the line would advance and stop; has not advanced for four or five days at this date, very black below the line.

Dr. Willier reported a parallel case in his own practice, which went on till the leg to the lower one-third of the thigh was dead, when the other foot began in the same way. At death, the flesh had all fallen off the legs; tried everything without relief.

Dr. Miller wanted the pathology of the disease. Dr. Chambers says the pathology is uncertain. If from a plug in an artery the remedy is amputation, if not, all the treatment necessary is opium to relieve pain, and food; advises not to amputate. Dr. Wallston would, as a last resort, use transfusion.

Dr. Geo. Kilner read an exceedingly instructive and valuable paper on the common diseases of the eye, which was ordered to be published by request of the Society.

Dr. J. L. Reit, from the committee on epidemics, read a report. Dr. Massie said the principal treatment of small-pox would be isolation and ventilation; would place his patient under shade trees; thinks the mortality in this disease should not be more than one in three hundred. Dr. Chambers would add to the treatment mur. tinc. iron; Dr. Miller, in petusses, uses brom. of amonium and veratrum.

Dr. M. W. Wilcox reported on practical medicine. The report was long, and prepared with great care; thinks if the practice of medicine could be established on some permanent basis, the task of reporting on it would be easy, but the great question regarding the treatment of disease, notwithstanding all the improvements, is but little nearer solution now than in the time of Hypocrates. Still he thinks the evidences of a steady improvement are most conclusive; ignorance within, and prejudice without, account for much of the failure.

Medicine now, is not only acknowledged to be a science, but a science necessary to the happiness of our race. The various systems of practice exert but little influence in disturbing the current of public and professional opinion. The great mass of intelligent men look for relief to the system represented by ours and kindred associations. It is a satisfaction to know that practical medicine is keeping pace with the march of mind. Though not equal to every emergency, we are yet accomplishing all that it is possible for the finite mind, when it enters the field to contend with the decree of fate. Mathematical certainty will never be arrived at in our profession. Our success will ever depend on our knowledge of auxiliary sciences and our power to adapt remedies so as to meet the emergencies of each individual case. We should profit by what has been done by others; the experience of everyone, however limited, is valuable. Although everything is changing, every change is not necessarily an improvement; the present time is productive of more that is of practical benefit than any previous period in the history of the sciences. He has used many of the new therapeutical agents with greater or less success, but is not disposed to abandon old-tried and proved remedies; the medical man, by their use, is in danger of pandering to the patent medicine system.

Subcutaneous medication is claiming considerable attention, and is growing in favor rapidly. Thinks nervous diseases are on the increase in his locality. If, as Prof. Aitken intimates, this is dependent upon extreme mental effort, there ought to be a corresponding increase of mental power,—this is not obvious where he resides.

Dr. Apperson read a carefully-prepared and elaborate paper upon "Gastric Juice." He objects to the term gastric juice, prefers "solvent." He reviewed at some length the five different theories, *viz.:* coction, putrefaction, trituration, fermentation, and chemical solution. After looking carefully over all the experiments and speculations of late authorities, he has the *courage* to say that there is yet error, and that the true source of the solvent of the stomach remains to be pointed out. His proposed theory differs in some respects from all preceding ones. All agree that digestion is performed in the stomach, but Dr. A. objects to the solvent being secreted by the coats of the stomach because of *its want of a glandular system.* Claims the glands of the mouth as furnishing the true solvent. Has arrived at this conclusion from observation on his own person through a long series of years, and from observation on ruminant animals; and the effects of medication in case of dyspepsia, claims that the saliva obtained from the parotids, sublingual and submaxillary glands furnishes the true solvent. The Doctor's conclusions were well drawn, and his arguments sustained by facts. Dyspepsia arises from a want of the true solvent, and is cured by its supply through the act of mastication of solid bodies without artificial diluents.

Dr. Chambers reported a case with unusual symptoms. Man, aged 38. For nearly three years past, every night when he goes to bed, and first falls to sleep, he ceases to breathe, and when aroused by his wife, who has to remain awake till after she arouses him from his first sleep, it is with great fright and most fearful sensations and apprehensions. The Doctor has treated the case for eighteen months, with but slight relief, from brom. potass. and Hall's solution of strychnine. Patient has had two attacks of intermittent fever; during the prevalence of the fever the unaccountable symptoms abated; no satisfactory diagnosis of the case.

Dr. Willien reported a case of calculus nephritis; treatment followed by recovery.

*Treatment.*—Externally, blister. Internally, with the following mixture:

℞. Potass. Acetatis, ---------- ℥ss.

Vini Colchici, ---------- ℥ss.

Spts. Nitroci Dulcis, ---------- ℥j.

Tinc. Opii Camph., ---------- ℥ss.

Fl. Ex. Belladonna, ---------- ʒj.

Aq. Cinnamomi, ---------- ℥ss.

M. Sig. One teaspoonful every three hours.

Next morning calculus passed the urethra. Weight, qrs. v. Phosphate lime. Immediate recovery.

The Society adjourned to meet in Paris, Ill., on the last Wednesday of Oct., 1871.

WM. M. CHAMBERS, *President.*

G. T. RAGAN, *Secretary.*

